# Correlation between Brodsky Tonsil Scale and Tonsil Volume in Adult Patients

**DOI:** 10.1155/2018/6434872

**Published:** 2018-10-24

**Authors:** Xiaotong Lu, Junbo Zhang, Shuifang Xiao

**Affiliations:** Department of Otorhinolaryngology, Head and Neck Surgery, Peking University First Hospital, No. 8 Xi Shiku Street, Xi Cheng District, Beijing 100034, China

## Abstract

**Purpose:**

To evaluate the value of Brodsky tonsil scale in predicting the objective tonsil volume and to identify the potential factors that might interfere with the accuracy of prediction.

**Methods:**

A total of 87 adult patients who underwent single tonsillectomy or uvulopalatopharyngoplasty (UPPP) procedure including tonsillectomy in our hospital between Jan 2015 and Dec 2016 were included. The data of Brodsky tonsil scale evaluated preoperatively and objective tonsil volume evaluated postoperatively were collected for analysis.

**Results:**

Among the 87 adult patients included, 85 patients underwent bilateral tonsillectomy, while only 2 underwent unilateral procedure. Therefore, a total of 172 tonsils were included. Significant positive correlations were established between Brodsky scale and objective volume for either right (R = 0.647), left (R = 0.664), or overall tonsils (R = 0.654) (all *p* < 0.001). However, volume overlaps could be found between 2+ and 3+ tonsils. Age [odds ratio (OR) = 4.053, *p* = 0.003] and body mass index (BMI; OR=1.740, *p* = 0.044) were found to be independent factors that could influence the consistency between the Brodsky scale and objective volume. As a result, a formula “Index = -1.409+1.399×age+0.554×BMI” was constructed for the evaluation of the consistency.

**Conclusion:**

Tonsil grading was significantly correlated with tonsil volume; preoperative tonsil grading that reflected the real tonsil volume was regarded as the protocol for the evaluation of the tonsil size. Age and BMI were independent factors that could affect the consistency between tonsil grade and tonsil volume. A mathematical model was estimated to predict the consistency accurately.

## 1. Introduction

Obstructive sleep apnea-hypopnea syndrome (OSAHS) presents repetitive partial or complete upper airway obstruction during sleep, resulting in hypoxemia and hypercapnia [1]. Patients may experience nocturnal symptoms such as snoring and apneas and daytime symptoms like sleepiness and impaired concentration [2], which increase the risks of cardiovascular diseases, hypertension, diabetes, and cognitive impairment [3, 4]. Continuous positive airway pressure (CPAP) is standard treatment for OSA; however, due to invasiveness, compliance is low that may affect treatment outcome [5]. As “salvage therapy,” surgery could improve the effectiveness and tolerance of CPAP [5]. Among various surgical procedures, uvulopalatopharyngoplasty (UPPP) is the most common surgical approach [6], which consists of removal of the tonsils, uvula, and part of the soft palate that aims to increase oropharyngeal area. Nevertheless, the success rate of UPPP was between 40 and 60% [7]. Despite modified procedures in recent years, the surgical outcome had little remarkable improvements [8]. Therefore, it is essential to find the outcome predictors for successful treatment. The evaluation of the tonsil size is valuable in clinical work, which is a major factor for the upper airway obstruction in OSAHS patients [9–12], as removal of big tonsils can significantly widen a relatively narrow pharynx. Thus, tonsil size is now commonly accepted as a predictive factor for successful surgical outcome [13–18]. Several methods and examinations can be employed for evaluating the tonsil size. The Brodsky scale [19] which is widely used in physical examination is a classical method. It is simple to be performed and costs little. However, its accuracy may be influenced by several subjective and objective factors theoretically, such as embedded tonsils or excessive oropharyngeal tissues. Clinically, we found that the tonsil grading may not reflect real tonsil volume, which may be underestimated or overestimated by subjective grading scale due to the embedded tonsils or excessive oropharyngeal tissues, leading to surgical chances missing or overtreatment. Recently, a study with a small-volume population showed that ultrasound could be used as a tool for tonsil volume assessment preoperatively in children with OSA and may help to prevent unnecessary surgical procedures [20]. However this method needs large volume cohort to evaluate reproducibility. This current study was performed to study the accuracy of Brodsky tonsil scale in reflecting the actual tonsil volume in adult patients. To our knowledge, this is the first research to study influence factors for correlation and establish a mathematical model to evaluate the consistency between tonsil grade and tonsil volume.

### 1.1. Patients and Methods

This is a prospective cohort study. The inclusion criteria consisted of patients with tonsillitis or OSAHS who received tonsillectomy or UPPP and we excluded adult patients without tonsillitis or OSAHS who underwent tonsillectomy or UPPP and children patients. A total of 87 adult patients, who underwent tonsillectomy alone or UPPP from 2015 to 2016 in our hospital, were recruited. Of these, 85 patients received bilateral tonsillectomy, and 2 underwent unilateral procedure. The information of patients was collected with respect to gender, age, height, weight, body mass index (BMI) [21], history of smoking and alcohol intake, apnea-hypopnea index (AHI), lowest oxygen saturation (LSAT), and other polysomnography data.

**Figure 1:**
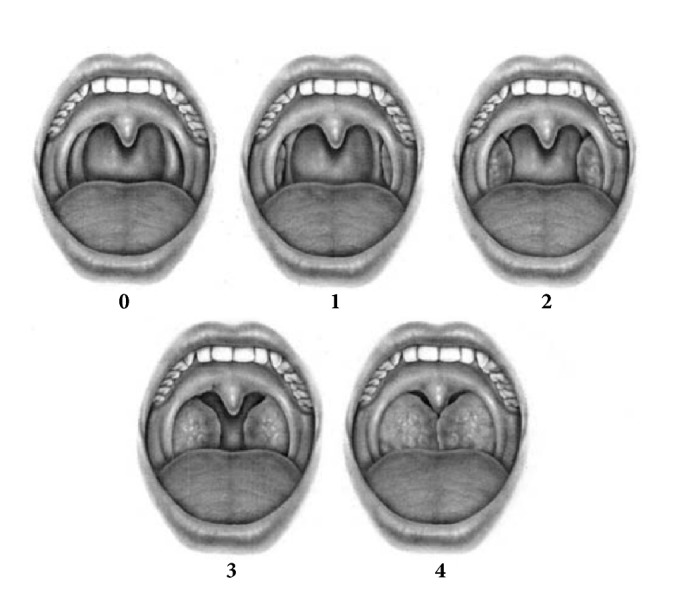
Brodsky grading scale for tonsil.

Two certified otolaryngologists classified palatine tonsils preoperatively by using Brodsky grading scale, classified into 5 grades as follows: grade 0 indicated the previous tonsillectomy; grade 1 indicated that the tonsils were hidden in the pillars; grade 2 indicated that the tonsils were beyond the anterior pillar and between 25 and 50% of the pharyngeal space; grade 3 indicated that the tonsils were beyond the pillars but not to the middle and occupied >50% and up to 75% of the pharyngeal space; grade 4 indicated that the tonsils occupied >75% of the pharyngeal space (Figure 1). Tonsil volume was measured postoperatively by water displacement based on Archimedes principle.

### 1.2. Statistical Analysis

Statistical analyses were conducted using SPSS software version 22.0. Spearman's rank correlation evaluated the correlation between tonsil grade and tonsil volume. Receiver operating characteristic curve (ROC curve) and Youden index were utilized to determine the cutoff value of tonsil volume. Binary logistic regression model analyzed the impact factors of the consistency between tonsil grade and tonsil volume and established the mathematical model including influence factors and consistency. A *p* value <0.05 was considered as significant.

## 2. Results

### 2.1. Baseline Clinical Information

The clinical characteristics of 172 tonsils were summarized in Table 1. Among 87 adult patients, 41 (47.1%) and 46 patients (52.9%) were diagnosed with OSAHS and chronic tonsillitis, respectively. The cohort comprised 54 male (62.1%) and 33 female patients (37.9%) with a median age of 35 years (Interquartile Range, IQR: 28–46 years). With respect to BMI, 33 patients (37.9%) were classified as normal weight (BMI <25 kg/m^2^), 23 (26.4%) as mild obesity (BMI ≥25 and <30 kg/m^2^), and 30 (34.5%) as moderate obesity (≥30 and <35 kg/m^2^), and 1 (1.2%) was classified as severe obesity (≥35 kg/m^2^). A total of 22 patients (25.3%) presented a history of smoking, and 14 (16.1%) had a history of alcohol intake. Among 172 tonsils, 51 (29.7%) were subjective grade I, 63 (36.6%) were subjective grade II, 55 (32.0%) were subjective grade III, and 3 (1.8%) were subjective grade IV. The mean ± standard deviation (SD) of the left objective tonsil volume was 4.5±2.6 mL, while that of the right one was 4.7±2.7 mL.

### 2.2. Correlation Analysis between Tonsil Grading and Tonsil Volume

**Figure 2:**
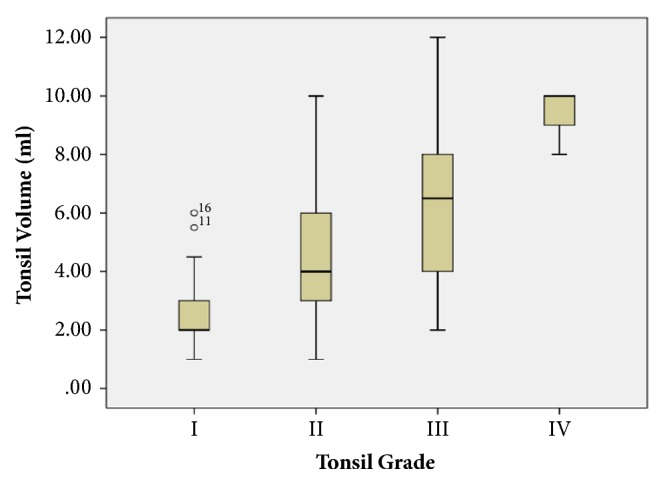
Box plot of tonsil grading and volume.

According to the postoperative measurements, the mean tonsil volume of grades 1, 2, 3, and 4 was 2.58±1.15, 4.33±1.99, 6.58±2.69, and 9.33±1.15 mL, respectively. Significant differences indicated that objective tonsil volume increased with subjective tonsil grade (Figure 2). Spearman's rank correlation analysis revealed significant associations between tonsil grades and tonsil volumes (*p* < 0.001, Table 2). Boxplot (Figure 2) revealed volume overlap between tonsil grades 2 and 3.

### 2.3. Analysis of the Consistency between Preoperative Tonsil Grade and Actual Tonsil Volume

We defined that tonsil grades 1 and 2 comprised the small tonsil group, while grades 3 and 4 comprised the big tonsil group. To assess the accuracy in predicting the small or big tonsil, we calculated the area under the ROC curve (AUC) for tonsil volume. The value of AUC was 0.833, and the optimal cutoff value of the tonsil volume was 6.25 mL. Then, we divided the whole group into small tonsil group (<6.25 mL) and big tonsil group (≥6.25 mL).  Table 3 shows that four new groups were created when actual tonsil volumes were compared: preoperatively big tonsil and actual tonsil volume ≥6.25 mL; preoperatively big tonsil but actual tonsil volume <6.25mL; preoperatively small tonsil and actual tonsil volume <6.25mL; preoperatively small tonsil but actual tonsil volume ≥6.25 mL. In binary logistic regression, after adjusting for other factors (Table 4), only age ≥35 years (*p* = 0.003) and increased BMI (*p* = 0.044) were identified as independent impact factors for the consistency between preoperative tonsil grade and actual tonsil volume.

A formula (Index=-1.409+1.399×age+0.554×BMI) had been created to evaluate the consistency. Age ≥35 years was set as 1, or as 0, and BMI <25 kg/m^2^ as 1, ≥25 and <30 kg/m^2^ as 2, ≥30 and <35 kg/m^2^ as 3, and ≥35 kg/m^2^ as 4. In order to assess the accuracy in predicting the consistency, we calculated the AUC for this index, which was 0.701, and the optimal cutoff value of tonsil volume was 2.22. Thus, when a patient's index is ≥2.22, his/her preoperative tonsil grade might accurately predict the actual tonsil volume.

## 3. Discussion

The narrow pharyngeal cavity plays a critical role in the pathophysiological process of some pharyngeal disease such as OSAHS; thus, the operation to enlarge the pharyngeal cavity could be regarded as an effective treatment. Current studies proved that tonsil size was one of the valuable predictors for surgical treatment; therefore, finding a precise method for assessing the tonsil size preoperatively could help in selecting the appropriate surgical candidates for a successful UPPP. In practice, we found that the tonsil grade could not reflect the real tonsil volume, and the correlation between them is not yet clearly established. Hitherto, only a few reports have been published about the correlation between the tonsil grade and tonsil volume. These studies reported a strong correlation between tonsil grade and tonsil volume in adult and children patients with sleep disorders, breathing, and chronic tonsillitis [12, 22–28], while discordance between tonsil grade and tonsil volume was also reported [23]. In this study, a significant correlation between the tonsil grade and tonsil volume was found in patients with chronic tonsillitis and OSAHS. Tonsil volumes increased with tonsil grades advanced; patients with tonsil grades 3 and 4 had a significantly greater volume than those with grades 1 and 2, which revealed that tonsil grade could represent the real tonsil size. Patients with greater tonsil grade were likely to benefit from tonsillectomy with additional changes in the pharyngeal cavity. Therefore, tonsil grade obtained via physical examination was conducive to select surgical candidates with a high success rate of treatment.

Nevertheless, we also found discordance between tonsil grade and tonsil volume in grade 2 and 3 groups, which indicated the limitation of the grading scale: the subjective tonsil grade may fail to predict the objective tonsil volume in patients with grades 2 or 3. This grading scale could be influenced by the subjective view of the examiner and the adjacent anatomical structures, thereby leading to underestimated or overestimated changes in the pharyngeal cavity via tonsillectomy in patients with OSAHS; for example, the objective tonsil volume in grade 3 may be ≤ that in 1 or 2, suggesting that some patients with large tonsil may lose the chance of surgery. Preoperative imaging examination such as magnetic resonance imaging (MRI) or computed tomography (CT) that offer multidimensional anatomical information about the upper airway may be more helpful in assessing the real tonsil size and improving the prediction accuracy.

In the present study, the univariate and multivariate analysis demonstrated that age and BMI affected the consistency between tonsil grade and tonsil volume. We speculated that pharyngeal cavity and embedded tonsil size decreased with age owing to tonsil atrophy and degeneration [26, 29], thereby decreasing the possibility of the embedded tonsil. In addition, palatine tonsils are lymphoid tissues that form a part of the Waldeyer ring. Increasing BMI revealed fat accumulation in peripheral tonsil space, which caused a decline in pharyngeal cavity, restricting the embedded tonsil growth. Taken together, tonsil grade scale and tonsil volume were highly consistent.

According to multivariate analysis, we established the following model about the consistency between tonsil grade and tonsil volume: Index=-1.409+1.399×age+0.554×BMI. 2.22 was determined as the cutoff value obtained from ROC curve and Youden index. Index with ≥2.22 prompted a greater consistency between tonsil grade and volume. Patients with grade 3 or 4 presented greater real tonsil volume and, thus, could benefit more from tonsillectomy and surgery. Patients with grade 1 or 2 might exhibit a failed UPPP due to small tonsil volume. Therefore, this model could not only identify the appropriate patients and select the therapeutic regime but also avoid unnecessary examination, preserve medical resources, and be cost-effective. In addition, it could be used in the follow-up of patients with chronic tonsillitis in order to evaluate the tonsil volume status. Index with <2.22 prompted that tonsil grades were not significantly correlated with tonsil volume, and grading scale failed to predict the tonsil volume accurately. However, we speculated that patients with grade 3 or 4 could undergo surgical treatment in order to expand visible upper airway since the tonsils grew to medial oropharyngeal cavity obstructing the upper airway. For patients with grade 1 or 2, the poor correlation indicated a greater tonsil volume. Thus, we recommended imaging examination for further evaluation or surgical treatment to reduce the obstruction of the upper airway. In this case, the physicians should inform the patients about the benefits and risks of imaging examination and surgery for appropriate clinical decision preferable to the patients. Briefly, we suggested surgery for patients with grade 3 or 4 despite the value of correlation. Operation was not recommended for those with grade 1 or 2 if the index was ≥2.22; conversely the patients with grade 1 or 2 with index with <2.22 should be informed of the benefits and risks. This mathematical model, characterized by noninvasion, high cost-efficiency, the simplicity of operation, and easy acquisition of data, could identify the suspected embedded tonsils, thereby providing a significant preoperative guide for appropriate surgical treatment.

Nevertheless, the present study had some limitations. Hitherto, a total of 87 adult patients with 172 tonsils were reported. Thus, further research with a large number of patients was essential to verify the accuracy and reproducibility of the model of correlation. In addition, all the patients in the current study were adults and children were not enrolled. Therefore, future studies should investigate the consistency between tonsil volume and tonsil grade in children. Subjective tonsil size was assessed by the Brodsky scale which is widely used in physical examination and easily performed. However, the accuracy of this subjective grading may be influenced by several factors, such as embedded tonsils or excessive oropharyngeal tissues. Hence, more comprehensive methods to evaluate the tonsil size preoperatively are necessary when treating patients receiving surgical treatment that could improve the narrow pharyngeal cavity.

## 4. Conclusion

Tonsil grading was significantly correlated with tonsil volume; thus preoperative tonsil grading by physical examination that reflected the real tonsil volume was regarded as the protocol for the evaluation of the tonsil size. However, the limitation of the grading scale that subjective tonsil grading may fail to predict the objective tonsil volume in patients with grade 2 or 3 leads to underestimated or overestimated changes in the pharyngeal cavity through tonsillectomy. Age and BMI independently affected the consistency between tonsil grade and tonsil volume, and the mathematical model may provide more accurate information of the tonsil size preoperatively that aided in selecting ideal candidate patients who expect to expand pharyngeal cavity through tonsillectomy.

## Figures and Tables

**Table 1:** Baseline characteristics.

Diagnosis	Chronic tonsillitis	46 (52.9%)
	OSAHS	41 (47.1%)
Median age	35 years (IQR: 28-46 y)	
Gender	Male	54 (62.1%)
	Female	33 (37.9%)
BMI (kg/m^2^)	<25	33(37.9%)
	≥25, <30	23(26.4%)
	≥30, <35	30(34.5%)
	≥35	1(1.2%)
Smoking	Yes	22(25.3%)
	No	65(74.7%)
Drinking	Yes	14(16.1%)
	No	73(83.9%)
Left tonsil grading	I	27(31.0%)
	II	33(37.9%)
	III	26(29.9%)
	IV	1(1.1%)
Right tonsil grading	I	24(28.2%)
	II	30(35.3%)
	III	29(34.1%)
	IV	2(2.4%)
Tonsil volume	Left	4.5±2.6ml
	Right	4.7±2.7ml

IQR= interquartile range; BMI= body mass index.

**Table 2:** The correlation between tonsil grading and tonsil volume.

	Left tonsil (n=87)		Right tonsil (n=85)		Bilateral tonsil (n=172)	
Tonsil volume	Coefficient	*P* value	Coefficient	*P* value	Coefficient	*P* value
	0.664	*P* < 0.001	0.647	*P* < 0.001	0.654	*P* < 0.001

By using Spearman's rank correlation.

**Table 3:** The consistency between preoperative tonsil grading and actual tonsil volume.

		Preoperative Tonsil Grading			
		Small tonsil	Big tonsil	Chi-square	*p*
		(n=114)	(n=58)		
Actual Tonsil volume	≥6.25 ml	6 (5.3%)	31 (46.6%)	42.266	<0.001
	<6.25 ml	108 (94.7%)	27 (53.4%)		

**Table 4:** Preoperative factors that predict the consistency between tonsil grading and volume in univariate and multivariate analyses.

Variables	Univariate		Multivariate	
	OR (95%CI)	*P* value	OR (95%CI)	*P* value
Gender (Male vs. Female)	2.014(0.935-4.337)	0.074	1.552(0.616-3.911)	0.351
Age (≥35 vs.<35 years)	3.724(1.613-8.601)	0.002^*∗*^	4.053(1.627-10.096)	0.003^*∗*^
BMI (1 vs.2 vs.3 vs.4)	1.727(1.056-2.826)	0.029^*∗*^	1.740(1.014-2.986)	0.044^*∗*^
Drinking (yes vs. no)	0.846(0.313-2.289)	0.742	0.069(0.102-1.089)	0.069
Smoking (yes vs. no)	1.693(0.648-4.422)	0.282	1.523(0.333-6.959)	0.587

By using binary logistic regression.

## Data Availability

Datasets are available from the corresponding author upon request.
